# Dietary resistant starch enhances immune health of the kidney in diabetes via promoting microbially-derived metabolites and dampening neutrophil recruitment

**DOI:** 10.1038/s41387-024-00305-2

**Published:** 2024-06-20

**Authors:** Matthew Snelson, Devy Deliyanti, Sih Min Tan, Anna M. Drake, Cassandra de Pasquale, Vinod Kumar, Trent M. Woodruff, Jennifer L. Wilkinson-Berka, Melinda T. Coughlan

**Affiliations:** 1 Department of Diabetes, School of Translational Medicine, Alfred Medical Research and Education Precinct, Monash University, Melbourne, VIC Australia; 2 Department of Anatomy and Physiology, School of Biomedical Sciences, University of Melbourne, Parkville, VIC Australia; 3 School of Biomedical Sciences, Faculty of Medicine, The University of Queensland, Brisbane, QLD Australia; 4 Baker Heart and Diabetes Institute, Alfred Medical Research and Education Precinct, Monash University, Melbourne, VIC Australia; 5 Drug Discovery Biology, Monash Institute of Pharmaceutical Sciences, Monash University Parkville Campus, Parkville, VIC Australia

**Keywords:** Diabetes complications, Nutrition

## Abstract

**Background:**

Dietary-resistant starch is emerging as a potential therapeutic tool to limit the negative effects of diabetes on the kidneys. However, its metabolic and immunomodulatory effects have not yet been fully elucidated.

**Methods:**

Six-week-old db/db mice were fed a diet containing 12.5% resistant starch or a control diet matched for equivalent regular starch for 10 weeks. db/m mice receiving the control diet were utilised as non-diabetic controls. Freshly collected kidneys were digested for flow cytometry analysis of immune cell populations. Kidney injury was determined by measuring albuminuria, histology, and immunohistochemistry. Portal vein plasma was collected for targeted analysis of microbially-derived metabolites. Intestinal histology and tight junction protein expression were assessed.

**Results:**

Resistant starch limited the development of albuminuria in db/db mice. Diabetic db/db mice displayed a decline in portal vein plasma levels of acetate, propionate, and butyrate, which was increased with resistant starch supplementation. Diabetic db/db mice receiving resistant starch had a microbially-derived metabolite profile similar to that of non-diabetic db/m mice. The intestinal permeability markers lipopolysaccharide and lipopolysaccharide binding protein were increased in db/db mice consuming the control diet, which was not seen in db/db mice receiving resistant starch supplementation. Diabetes was associated with an increase in the kidney neutrophil population, neutrophil activation, number of C5aR1+ neutrophils, and urinary complement C5a excretion, all of which were reduced with resistant starch. These pro-inflammatory changes appear independent of fibrotic changes in the kidney.

**Conclusions:**

Resistant starch supplementation in diabetes promotes beneficial circulating microbially-derived metabolites and improves intestinal permeability, accompanied by a modulation in the inflammatory profile of the kidney including neutrophil infiltration, complement activation, and albuminuria. These findings indicate that resistant starch can regulate immune and inflammatory responses in the kidney and support the therapeutic potential of resistant starch supplementation in diabetes on kidney health.

## Introduction

Diet plays a crucial role in the development of diabetes and its associated complications, including diabetic kidney disease (DKD). Interventions utilizing dietary fiber have been demonstrated to improve markers of renal health in people with chronic kidney disease [1]. Despite this, people with and at risk of developing kidney disease have inadequate intake of dietary fiber, which has been identified as a significant concern [2]. Of particular importance is fermentable fiber, which can be utilized by the gut microbiota. Resistant starch (RS), a type of fermentable fiber, offers a promising dietary therapeutic option as it maintains many of the organoleptic and cooking properties of regular starch [3]. There are five different types of RS, which are classified depending on the mechanism by which they are resistant to digestion by α-amylases, with resistant starch type 2 being the most widely studied [4]. Moreover, RS exhibits a slower fermentation rate compared to other fermentable fibers like inulin, fructo-oligosaccharides, and galacto-oligosaccharides, reducing gastrointestinal discomfort associated with their consumption [5].

Several studies have indicated that RS may be a promising therapeutic tool to protect against the development of diabetic kidney disease, however, the underlying mechanisms are still being investigated [3]. Whilst the effects of RS on the gut microbiome have been well studied [3], there has been a dearth of understanding about the effects of RS on the production of microbially-derived metabolites in diabetes. Much of the focus has been on short chain fatty acids (SCFAs) which are produced by microbial fermentation of non-digestible carbohydrates that are available to saccharolytic bacteria in the colon. These SCFAs exhibit immunomodulatory properties and are able to act on a number of receptors present in immune cells [6]. This study aimed to investigate the effects of RS supplementation on microbially-derived metabolites and immune cells in the kidney using an experimental model of diabetes. By examining the influence of RS on these parameters, we provide insight into the potential value of using RS as a diet therapy for managing DKD.

## Methods

### Animals

4-week old male db/m and db/db mice either heterozygous or homozygous for a mutation in the leptin receptor gene (*Lepr)* gene (BKS. Cg-Dock7^m^ + /+ Lepr^db^/J) respectively, were purchased from The Jackson Laboratory (Bar Harbor, ME). The db/db mouse exhibits chronic hyperglycemia, hyperphagia, obesity, and insulin resistance and is widely utilized as a model of type 2 diabetes mellitus, whilst the db/m mouse is a suitable non-diabetic control mouse [7]. The number of mice per group was selected based on pilot data, to detect a change in the urinary albumin creatinine ratio of at least 100 g/mol (α = 0.05, 1 - β = 0.8) between the control and RS-supplemented diabetic mice. Mice were acclimatized for a period of 2 weeks, prior to the commencement of diet intervention at 6 weeks of age. Mice were housed in a climate-controlled animal facility with a 12:12 h light-dark cycle and received *ad libitum* access to mouse chow and water. Each week, mice were weighed and had random blood glucose levels measured using an Accu-Chek® Performa glucometer (Roche Diagnostics, USA). All study protocols were conducted in accordance with the principles and guidelines devised by the Alfred Medical Research & Education Precinct Animal Ethics Committee (AMREP AEC) under the guidelines laid down by the National Health and Medical Research Council of Australia and had been approved by the AMREP AEC (E1487/2014/B).

### Diets

**Fig. 1:**
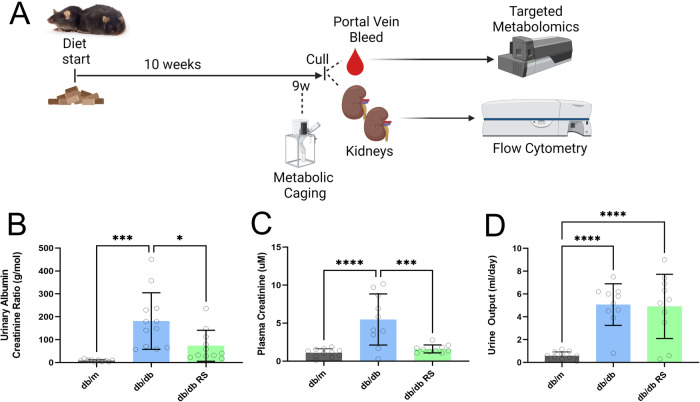
Resistant starch reduces kidney injury in diabetic mice. **A** Schematic of study design for Cohort 1. Created with biorender. **B** Urinary Albumin Creatinine Ratio, **C** Plasma Creatinine Levels, **D** 24-h urine output. * = *P* < 0.05, *** = *P* < 0.001, **** = *P* < 0.0001. One-way ANOVA with Tukey’s post hoc test. Data = mean ± SD. *n* = 10–12.

At 6 weeks of age, mice commenced a specialty formulated semi-pure resistant starch supplemented diet (SF15-015) containing 25% g/g Hi-maize 1043 (Ingredion, Westchester, IL), equivalent to 12.5% g/g resistant starch type 2 [8] or a custom-made matched control diet (SF15-021). Both diets were prepared by Specialty Feeds (Perth, Western Australia, Australia) and were matched in terms of total caloric content, protein and fat (Supplementary Table 1). The control diet was received by the non-diabetic mice (designated “db/m”). The diabetic mice were randomly allocated by cage to receive either the control diet (designated “db/db”) or the resistant starch supplemented diet (designated “db/db RS”). Mice received these diets *ad libitum* for a period of 10 weeks. Two cohorts of mice, referred to in this paper as Cohort 1 and Cohort 2, were utilized for different experimental endpoints. Both cohorts received the same diets. The experimental overview for each cohort is demonstrated in Fig. 1A and Fig. 4A, respectively.

### Metabolic caging, plasma and tissue collection

At experimental week 9 (15 weeks of age), mice in Cohort 1 were housed individually in metabolic cages (Iffa Credo, L’Arbresle, France) for 24 h for urine collection and measurement of water and food intake. Commercially available ELISA tests were utilized for the measurement of urinary albumin (Kit: E90-134, Bethyl Laboratories Inc., USA) and C5a (Kit: DY-1250, R&D Systems, USA). Urinary creatinine was determined via a commercially available assay kit (Kit: 03263991190, Roche Diagnostics Corporation, USA) by a Cobas Integra 400 Plus autoanalyzer (Roche Diagnostics Corporation, USA). At the end of the experimental intervention, mice were anesthetized by intraperitoneal injection of sodium pentobarbitone (100 mg/kg body weight; Euthatal; Sigma-Aldrich, Castle Hill, Australia). Following euthanasia, blood was drawn from the portal vein, treated with sodium citrate (3.2% v/v), centrifuged at 6000 rpm for 6 mins, and plasma was collected and stored at −80 °C. Both kidneys were removed for tissue digestion and flow cytometry.

As both kidneys were utilized in Cohort 1 for flow cytometry to investigate renal immune cell populations, a second cohort of mice was run to investigate the effects of these diets on renal and intestinal histology. At the endpoint, mice were anesthetized by intraperitoneal injection of sodium pentobarbitone (100 mg/kg body weight; Euthatal; Sigma-Aldrich, Castle Hill, Australia) followed by cardiac exsanguination. Cardiac blood was centrifuged at 6000 rpm for 6 mins, and plasma was collected and stored at −80 °C. Kidney sections were fixed in neutral buffered formalin (10% v/v) before being embedded in paraffin. The gastrointestinal tract was dissected, and the mesentery was removed. The total gastrointestinal tract, and then the dissected cecum and colon, were weighed and length was measured. The gastrointestinal tract was flushed with chilled phosphate-buffered saline. Jejunum and ileum sections were fixed in paraformaldehyde (4% v/v) for 24 h before being transferred to 4% sucrose solution and embedded in paraffin. Jejunum, ileum, and colon sections were snap-frozen in liquid nitrogen and stored at −80 °C.

### Flow cytometry

Both kidneys were collected from mice in Cohort 1. A single-cell suspension was prepared as follows. Briefly, both kidneys from each mouse were mechanically dissociated (sliced into cubes ~1–2 mm, by scissors) followed by orbital incubation at 37 °C with Collagenase IV (1 mg/ml, Sigma-Aldrich) for 30 min in RPMI 1640 medium with 5% fetal bovine serum. Kidney homogenates were filtered through a 70-μm cell strainer, and then subjected to a Percoll gradient centrifugation in 36% Percoll (Sigma-Aldrich), overlaid onto a 72% Percoll solution and centrifuged at 1000 g with no brake for 20 min at 4 °C. Cells were isolated from the Percoll interface and washed in cold media (RPMI 1640 medium with 5% fetal bovine serum). Erythrocytes were lysed using RBC lysis buffer (BD Biosciences). The resulting single-cell suspension was then treated with Fc Block (anti-CD16/CD32, BD Biosciences, clone 2.4G2) to block non-specific binding of antibodies. Cells were analysed with a panel of antibodies including Brilliant Violet 786–conjugated (BV786) anti-CD45 antibody (BD Biosciences, clone 30-F11, 1/2000), AF700-conjugated anti-CD11b antibody (Biolegend, clone M1/70, 1/1000), BV605-conjugated anti-Ly6C antibody (BD Biosciences, clone AL-21, 1/1000), Fluorescein isothiocyanate-conjugated (FITC) anti-C5aR1 antibody (Cedarlane, clone 20/70, 1/300), PE anti-Ly6G antibody (Tonbo biosciences, clone 1A8, 1/1000). To distinguish between live and dead cells, cells were stained with a Fixable Aqua live dead cell stain kit (Invitrogen, L34966). All antibody and live dead stain concentrations were used according to the manufacturer’s recommendations. Cells were counted with a Tali Image-Based Cytometer (Life Technologies). Analysis was performed on a BD FACSCanto II system using BD FACSDiva software (BD Biosciences).

### Plasma analyses and targeted metabolomics

At cull, portal vein blood was collected from mice in Cohort 1. Plasma lipopolysaccharide-binding protein (LBP) was measured using an ELISA (ab269542, Abcam) as per the manufacturer’s instructions. The intra-assay coefficient of variation was 5.2%. Plasma endotoxin was measured using a commercially available assay based on Limulus amoebocyte lysate (A39552, Pierce Thermo Scientific, Rockford, IL, USA). Portal vein blood that had not undergone any prior freeze-thaw cycles was used for targeted metabolomics as previously described [9].

### Renal histology

Kidney sections with a thickness of 3 micrometers were subjected to periodic-acid Schiff (PAS) staining. The extent of sclerosis in each glomerulus was evaluated using a subjective 0–4 scale, where 0 represented normal conditions, and higher grades indicated increased sclerotic areas: minimal (grade 1), sclerotic area up to 25%; moderate (grade 2), sclerotic area 26 to 50%; grade 3, sclerotic area 51 to 75%; severe (grade 4), sclerotic area 76 to 100% (severe). The Glomerulosclerosis Index (GSI) was computed using a formula that factored in the number of glomeruli in each grade as previously described [10]. Digital images of the renal cortex were taken with an Eclipse Ci brightfield microscope (Nikon, Melville, NY, USA) at 200x magnification. Evaluations were performed using Image-Pro Plus (version 7.0; Media Cybernetics, Bethesda, MD, USA) and analysis was conducted in a blinded fashion.

### Renal immunohistochemistry

Paraffin sections of mouse kidney (4 μm) were immunostained with rabbit anti-fibronectin (AO245, DAKO) at a 1:400 dilution. In brief, endogenous peroxidases were blocked by 3% hydrogen peroxide for 15 min, followed by a pepsin antigen retrieval for 10 min, after which samples were blocked in Dako Superblock (1:10 in TBS) for 20 min. A primary antibody was applied and left overnight at 4 °C. The following day, slides were incubated with biotinylated secondary antibody for 10 min at room temperature. Sections then underwent incubation with Vectastain ABC reagent (Vector Laboratories, CA, USA). Peroxidase activity was identified by reaction with 3,3’-diaminobenzidine tetrahydrochloride (Sigma-Aldrich Pty. Ltd, NSW, Australia). Counterstaining with hematoxylin was done to identify nuclei. Sections were examined under light microscopy (Olympus BX-50; Olympus Optical). All digital quantitation (Image-Pro Plus, v7.0) and assessments were performed in a blinded manner.

### Intestinal Histology

Paraffin-embedded ileum and jejunum sections, with a thickness of 5 micrometers, were prepared for analysis. To assess villi length and crypt depth, the sections were stained with hematoxylin and eosin. For the enumeration of goblet cells, the sections were initially stained with 1% Alcian blue for 15 min, followed by periodic acid Schiff staining. Images were captured using an Eclipse Ci brightfield microscope (Nikon, Melville, NY, USA) at a 100x magnification. Image-Pro Plus software (version 7.0, Media Cybernetics, Bethesda, MD, USA) was employed to view the images and measure villi length and crypt depth utilizing the built-in measurement tool. The number of goblet cells per villus was manually counted for each villus. All analyses were performed in a blinded manner.

### Quantitative RT-PCR

RNA was extracted from snap-frozen colon, jejunum and ileum samples using TRIzol Reagent (Life Technologies) as previously described [10]. cDNA was synthesized from RNA using M-MuLV Reverse Transcriptase (Thermo Fischer). Expression of zonula occludens-1 (ZO-1; *Tjp1*) and junctional adhesion molecule A (JAM-A; *F11r*) was determined using TaqMan reagents (Life Technologies, Carlsbad, CA). For occludin (*Ocln*), claudin-1 (*Cldn1*), claudin-2 (*Cldn2*), claudin-3 (*Cldn3*), claudin-4 (*Cldn4*), claudin-5 (*Cldn5*) and claudin-7 (*Cldn7*), SYBR Green reagents (Applied Biosystems, California, USA) were utilized. RT-PCR was conducted using either a QuantStudio 3 or 5 Real-Time PCR System (Thermo Fisher). Gene expression was normalized to *β*-actin (Applied Biosystems) and fold change was calculated relative to db/m CON mice using the ΔΔCT method.

### Statistical analysis

Statistical analyses were performed using Graphpad Prism (Version 9.3.1, Graphpad Software, USA). Outliers were assessed and removed using robust regression and outlier removal (ROUT) [11] using a false discovery rate (FDR) value of 0.01. Data were assessed for normality using Kolmogorov-Smirnov test. Normally distributed data were analyzed using one-way ANOVA with Tukey’s post hoc test for multiple comparisons. Non-normally distributed data were assessed using Kruskal-Wallis test with Dunn’s multiple comparisons test. Targeted metabolomics data were log-transformed and analyzed using Metabolanalyst (version 5.0) [12], with a fold change threshold of 2 and an FDR threshold of 0.05.

## Results

### Resistant starch supplementation improves kidney function in the db/db mouse

An overview of the study protocol for Cohort 1 is provided in Fig. 1A. Diabetic db/db mice had increased albuminuria compared with non-diabetic mice (Fig. 1B, *p* < 0.001). Supplementation of RS led to a decrease in the urinary albumin creatinine ratio (Fig. 1B, *p* < 0.05), indicative of a nephroprotective effect of RS in the context of DKD. Similarly, increased plasma creatinine levels indicated that kidney function was impaired in the db/db mice compared to db/m mice, an effect that was alleviated with RS supplementation (Fig. 1C, *p* < 0.001). The effect of RS on kidney injury and function in db/db mice was independent of any effect on urine output (Fig. 1D).

### Resistant starch supplementation promotes a non-diabetic plasma metabolite profile in diabetic mice and reduces intestinal permeability

**Fig. 2:**
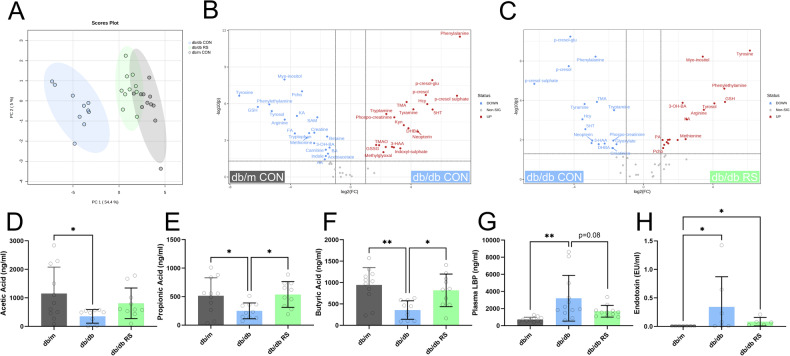
Resistant starch alters the plasma metabolite profile of db/db mice to be more similar to that of db/m mice. Targeted metabolomics analysis was conducted on portal vein plasma. **A** Principal components analysis plot, **B** volcano plot contrasting db/m CON and db/db CON, **C** volcano plot contrasting db/db CON and db/db RS. Portal vein plasma concentrations of the short chain fatty acids **D** Acetic Acid, **E** Propionic Acid and **F** Butyric Acid. Portal vein plasma concentrations of **G** Lipopolysaccharide Binding Protein (LBP) and **H** Endotoxin. * = *P* < 0.05, ** = *P* < 0.01. One-way ANOVA with Tukey’s post hoc test. Data = mean ± SD. *n* = 5–12.

Targeted metabolomics analysis of portal vein plasma revealed that samples from db/db mice consuming the RS diet clustered more closely with samples from db/m mice than the db/db mice on the control diet (Fig. 2A). Differential analysis between db/m mice and db/db mice both receiving control diets revealed that several uremic toxins including p-cresol sulfate, p-cresol glucuronide and indoxyl sulfate were increased in db/db mice compared to db/m (Fig. 2B). These same metabolites were decreased in db/db mice receiving a control diet relative to db/db mice receiving RS (Fig. 2C). The short chain fatty acids acetate (Fig. 2D), propionate (Fig. 2E) and butyrate (Fig. 2F) were reduced with diabetes. In diabetic mice concentrations of propionate and butyrate significantly increased with RS supplementation (Fig. 2D, E, *p* < 0.05).

Intestinal permeability was increased in diabetes as assessed by measurement of plasma lipopolysaccharide binding protein (Fig. 2G, *p* < 0.01) and plasma endotoxin (Fig. 2H, *p* < 0.05). Between diabetic mice cohorts, supplementation with RS led to a 47% reduction in plasma concentrations on lipopolysaccharide binding protein (Fig. 2G, *p* = 0.08) and a 75% reduction in plasma endotoxin (Fig. 2H, n.s.)

### Resistant starch alters neutrophil infiltration and activation in the diabetic kidney

**Fig. 3:**
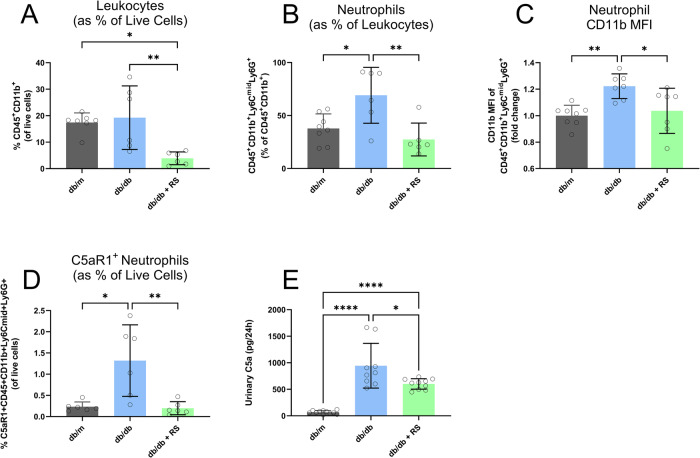
Resistant starch reduces neutrophil infiltration and complement activation in diabetic mice. Flow cytometry of kidneys showing **A** Leukocytes, **B** Neutrophils, **C** Mean Fluorescence Intensity (MFI) of CD11b on Neutrophils Cd11b **D** Percentage of C5aR1 positive Neutrophils **E**) 24-h urinary C5a excretion. * = *P* < 0.05, ** = *P* < 0.01, **** = *P* < 0.0001. One-way ANOVA with Tukey’s post hoc test. Data = mean ± SD. Flow cytometry data: n 4–5, urinary c5a data: *n* = 10–12.

The flow cytometry gating strategy is demonstrated in Supplementary Fig. 1. Whilst there was no discernible difference in total leukocyte populations between db/m and db/db mice, RS led to a noticeable decrease in leukocytes in the diabetic kidney (Fig. 3A). Diabetes led to an increase in kidney neutrophils which was reversed with RS supplementation (Fig. 3B). Neutrophils in diabetic mice exhibited greater mean fluorescence intensity (MFI) of the activation marker CD11b compared with non-diabetic mice (Fig. 3C). CD11b was reduced in diabetic mice consuming RS (Fig. 3C). The proportion of C5aR1+ neutrophils was increased in diabetes, which was not observed in db/db mice fed an RS supplemented diet (Fig. 3D). Diabetes led to an increase in urinary complement cascade activation component C5a (Fig. 3E, *p* < 0.0001) which was ameliorated with RS supplementation (Fig. 3E, *p* < 0.05).

### Resistant starch does not influence glomerulosclerosis or fibrosis

**Fig. 4:**
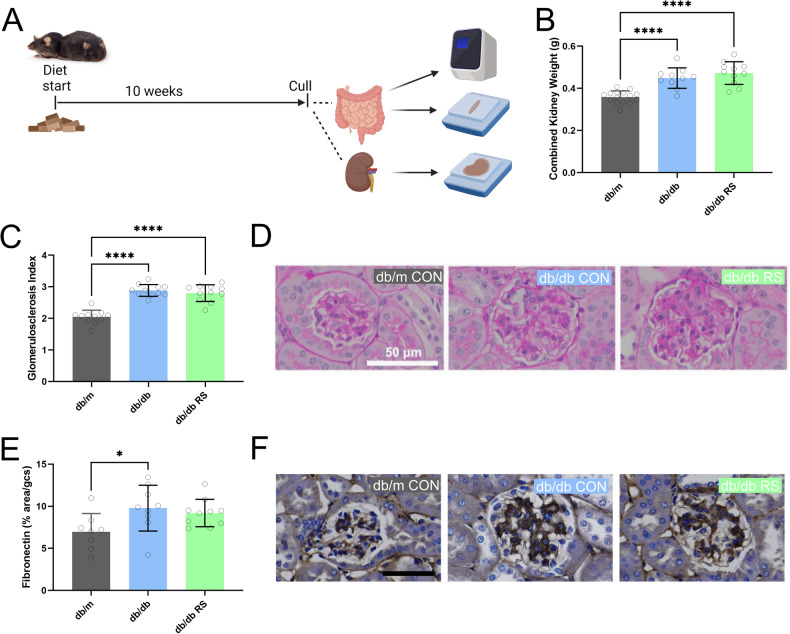
Resistant starch supplementation doesn’t alter renal fibrosis in diabetic mice. **A** Schematic of study design for Cohort 2. Created with biorender. **B** Weight of both kidneys combined. **C** Glomerulosclerosis Index and **D** representative images of Periodic acid-Schiff (PAS) stained kidney sections. **E** Fibronectin staining per glomeruli area and **F** representative images of Fibronectin deposition as examined by immunohistochemistry. * = *P* < 0.05, *** = *P* < 0.001, **** = *P* < 0.0001. One-way ANOVA with Tukey’s post hoc test. Data = mean ± SD. *n* = 11–16.

An overview of the study protocol for Cohort 2 is provided in Fig. 4A. Diabetic mice had an increase in relative kidney weight compared with non-diabetic mice (Fig. 4B, *p* < 0.01). Diabetes was associated with an increase in glomerulosclerotic index (Fig. 4C, *p* < 0.0001 and D) and glomerular fibronectin deposition (Fig. 4E, *p* < 0.05 and F). Resistant starch supplementation did not affect the degree of glomerulosclerosis or glomerular fibrosis.

### Diabetes promotes villi height, which is not affected by resistant starch supplementation

**Fig. 5:**
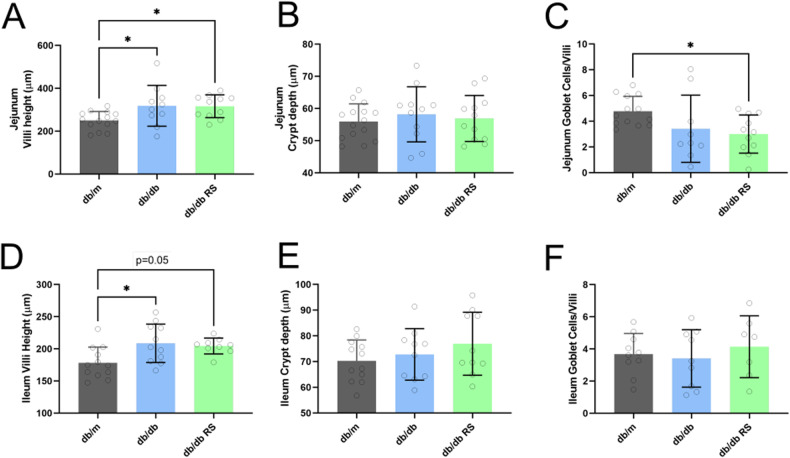
Diabetes increases villi height in both Jejunum and Ileum, with no effect of resistant starch. **A** villi height, **B** crypt depth and **C** number of goblet cells per villi in the jejunum. **D** villi height, **E** crypt depth and **F** number of goblet cells per villi in the ileum. * = *P* < 0.05. One-way ANOVA with Tukey’s post hoc test. Data = mean ± SD. *n* = 11–16.

Diabetes increased villi height in the jejunum which was not affected by RS supplementation (Fig. 5A, *p* < 0.05). There were no differences in jejunum crypt depth between groups (Fig. 5B). There was no effect of diabetes on the number of goblets cells per villi in the jejunum (Fig. 5C). In the ileum, diabetes increased villi height, which was not altered by RS supplementation (Fig. 5D, *p* < 0.05). There were no differences in crypt depth (Fig. 5E) or goblets cells per villi (Fig. 5F) in the ileum. Resistant starch supplementation led to an increase in the length and weight of the cecum and colon, but not the small intestines (Supplementary Fig. 2).

### Diabetes alters expression of tight junction proteins in jejunum and ileum, which is ameliorated with resistant starch

**Fig. 6:**
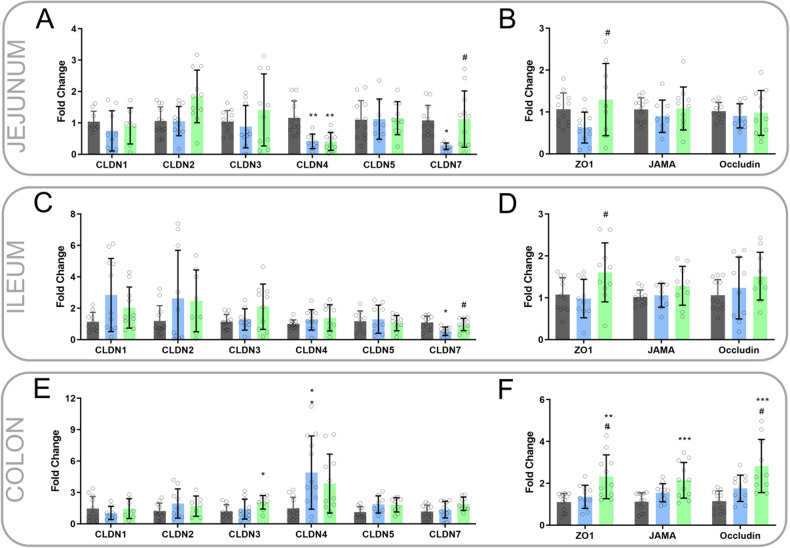
Intestinal Expression of tight junction proteins. Gene expression of **A** claudins (CLDN) and **B** tight junction proteins Zonula occludens-1 (ZO-1), JAMA (Junctional Adhesion Molecule A) and occludin in the jejunum. Gene expression of **C** claudins and **D** ZO-1, JAMA, and occludin in the ileum. Gene expression of **E** claudins and **F** ZO-1, JAMA and occludin in the colon. One-way ANOVA with Tukey’s post hoc test. Significantly different compared with db/m CON * = *P* < 0.05, ** = *P* < 0.001, *** = *P* < 0.001, **** = *P* < 0.0001. Significantly different compared with db/db CON # = *P* < 0.05, ## = *P* < 0.001, ### = *P* < 0.001, #### = *P* < 0.0001. Data = mean ± SD. *n* = 7–12.

In the jejunum, diabetes was associated with a decrease in gene expression of claudin 4 and claudin 7, with resistant starch ameliorating the diabetes-induced decrease in claudin 7 (Fig. 6A). Between diabetic mice, RS increased jejunum expression of ZO-1 (Fig. 6B). In the ileum, diabetes was associated with a decrease in gene expression of claudin 7 which was reversed by resistant starch (Fig. 6C). Between diabetic mice, RS increased ileum expression of ZO-1 (Fig. 6D). There was no effect of diabetes on claudin-7 expression in the colon (Fig. 6E). Between diabetic mice, RS increased jejunum expression of ZO-1 and Occludin (Fig. 6F).

## Discussion

This study demonstrates that RS prevents the decline in renal function seen in diabetes and provides insights into the mechanisms of action. Notably, we demonstrated a substantial shift in microbially-derived metabolites entering the systemic circulation, measured in portal vein plasma. The metabolomic profile of db/db mice receiving RS supplementation clustered closely with that of non-diabetic db/m mice. Of particular relevance is the rescue effect that RS had on increasing SCFAs levels to that seen in db/m mice. Diabetes has been associated with increased intestinal permeability in large human cohorts [13], which we also observed in this mouse model. Of relevance is that supplementation with RS reduced this intestinal permeability providing insight into the potential nephroprotective mechanism of action.

RS is a potent promoter of microbial butyrate production, with in vitro studies indicating that it is capable of producing twice as much butyrate compared with other fermentable fibers [14]. Butyrate is a key energy source for intestinal epithelial cells and plays a crucial role in the maintenance of the integrity of the intestinal barrier [15]. In line with these findings, our study confirmed that RS supplementation led to a significant increase in butyrate levels. This increase further supports a previous study conducted in a nephrectomized rat model, which demonstrated the effectiveness of butyrate supplementation in reducing intestinal permeability and proteinuria [16]. The observed elevation in butyrate levels following RS supplementation in our study provides additional evidence for the protective effects of butyrate in reducing intestinal permeability. Without protection against increased permeability of the intestinal tract, the translocation of pro-inflammatory microbial components, such as lipopolysaccharide, leads to the activation of systemic inflammatory responses that can have detrimental actions in tissues.

We observed notable improvements in the urinary albumin creatinine ratio and plasma creatinine in diabetic mice receiving RS, with several plausible mechanisms by which this may occur. RS has been shown in vivo to increase SCFA levels [17] and SCFAs have been shown to stimulate the secretion of GLP-1 in a GPR43-dependent manner [18]. Indeed, several animal studies demonstrate that RS supplementation increases circulating GLP-1 [19, 20]. Treatment of diabetic mice with GLP-1 receptor agonists inhibits NAD(P)H oxidase activity, reducing renal oxidative stress [21], and promotes the resolution of inflammation via attenuating production of bone marrow-derived progenitor cells and kidney inflammation [22] and thus RS may limit renal oxidative stress, inflammation, and subsequent kidney injury via a GLP-1-mediated mechanism. In the current study, RS supplementation was also associated with a reduction in the portal plasma concentration of p-cresol sulfate, a known uremic toxin [23]. In vitro, p cresol sulfate has been shown to increase inflammatory gene expression [24] and impair mitochondrial function [25] in renal proximal tubular cells, with in vivo animal work identifying that p cresol sulfate enhances NAD(P)H oxidase activity, oxidative stress, and subsequent renal fibrosis [26]. In people with chronic kidney disease, p-cresol sulfate levels are predictive for the progression of renal disease [27] and correlate with inflammatory markers [28] and mortality [29]. Increasing prebiotic dietary fiber intake decreases plasma p cresol sulfate levels [30, 31], providing further evidence that microbiota-targeted dietary modulation reduces this uremic toxin, which may have subsequent benefits in terms of reduced renal oxidative stress and lessened kidney injury.

Neutrophils act as a first line of defense against invading pathogens and recently have been increasingly recognized to play a pivotal role in the low-grade inflammation that can occur in chronic non-infectious conditions such as diabetes. While systemic neutrophil populations have been associated with worsened diabetic nephropathy [32], little is known about neutrophil populations within the kidney in the context of diabetes. Here, we have demonstrated that there is increased renal neutrophil infiltration in the setting of diabetes and showed that dietary RS supplementation can reduce both neutrophil infiltration and activation.

The complement cascade is an essential immune response mechanism that produces complement components like C5a, which can act on the C5aR1 receptor present on several myeloid-derived cells including neutrophils [33]. Previous studies have shown that genetic deletion of the receptor for C5a reduces the number of glomerular neutrophils in a model of antineutrophilic cytoplasmic antibody (ANCA) vasculitis [34]. Additionally, pharmacological inhibition of C5a signaling has been demonstrated to ameliorate diabetic nephropathy in animal models [35, 36], although neither of these studies investigated the effect on renal neutrophil populations. In this study, we noted that diabetes was associated with an increase in the number of C5aR1+ neutrophils in the kidney, which supports the involvement of the complement pathway in the activation of pro-inflammatory neutrophils. Notably, resistant starch supplementation was associated with a decrease in the number of C5aR1+ neutrophils, suggesting that resistant starch can downregulate pro-inflammatory neutrophil infiltration and activation within the kidney by modulating the complement cascade.

Furthermore, we found that diabetic mice supplemented with resistant starch exhibited a reduction in CD11b expression levels in neutrophils. CD11b is an activation marker that plays a critical role in neutrophil adhesion and migration to sites of inflammation [37]. The decrease in CD11b expression implies a dampening of neutrophil activation, which aligns with the overall decrease in renal neutrophil infiltration and the decrease in the number of C5aR1+ neutrophils observed in diabetic mice supplemented with resistant starch. Overall, our findings indicate that resistant starch has the potential to modulate the activation state of neutrophils, potentially reducing their inflammatory response in the context of diabetic kidney disease. By downregulating pro-inflammatory neutrophil infiltration and activation within the kidney through modulation of the complement cascade, resistant starch may contribute to the attenuation of renal inflammation in diabetic mice. This attenuation of renal inflammation may be the mechanism by which resistant starch, through the action of microbially produced metabolites such as butyrate, lead to the reduction of albuminuria and plasma creatinine.

In summary, this study demonstrates that dietary resistant starch can reduce diabetic nephropathy and suggests this may occur via alterations of the microbially-produced metabolites which have immunomodulatory effects. These findings indicate that diet is a robust non-pharmacological approach to target pro-inflammatory and immune responses both systemically and in the kidney, to improve markers of diabetic kidney disease and subsequent progression to end stage renal disease.

### Supplementary information

Supplementary Figure and Table Legends

Supplementary Table 1: Macronutrient composition of diets.

Supplementary Figure 1: Gating Strategy for kidney flow cytometry.

Supplementary Figure 2: Resistant starch increases large intestinal, but not small intestinal, weight and length in diabetic mice.

## Data Availability

The datasets generated during and/or analyzed during the current study are available from the corresponding author on reasonable request.
