# Supplementary material for: Dietary resistant starch enhances immune health of the kidney in diabetes via promoting microbially-derived metabolites and dampening neutrophil recruitment
Source: Nutr Diabetes. 2024 Jun 20;14:46. doi: 10.1038/s41387-024-00305-2 (PMC11190267; doi:10.1038/s41387-024-00305-2)
Supplement: Supplementary file 1 — Supplementary Figure and Table Legends [file 41387_2024_305_MOESM1_ESM.docx]

# Supplementary Figure and Table Legends

**Supplementary Table 1: Macronutrient composition of diets.**

**Supplementary Figure 1: Gating Strategy for kidney flow cytometry.**

**Supplementary Figure 2: Resistant starch increases large intestinal, but not small intestinal, weight and length in diabetic mice.**

Length of the A) small intestine, B) cecum and C) colon. Relative weight (normalised to body weight) of the D) small intestine, E) cecum and F) colon. * = P<0.05, *** = P<0.001, **** = P<0.0001. One-way ANOVA with Tukey’s post hoc test. Data = mean ± SD. n=11–16.
