# Supplementary figures and images for: Dietary resistant starch enhances immune health of the kidney in diabetes via promoting microbially-derived metabolites and dampening neutrophil recruitment
Source: Nutr Diabetes. 2024 Jun 20;14:46. doi: 10.1038/s41387-024-00305-2 (PMC11190267; doi:10.1038/s41387-024-00305-2)

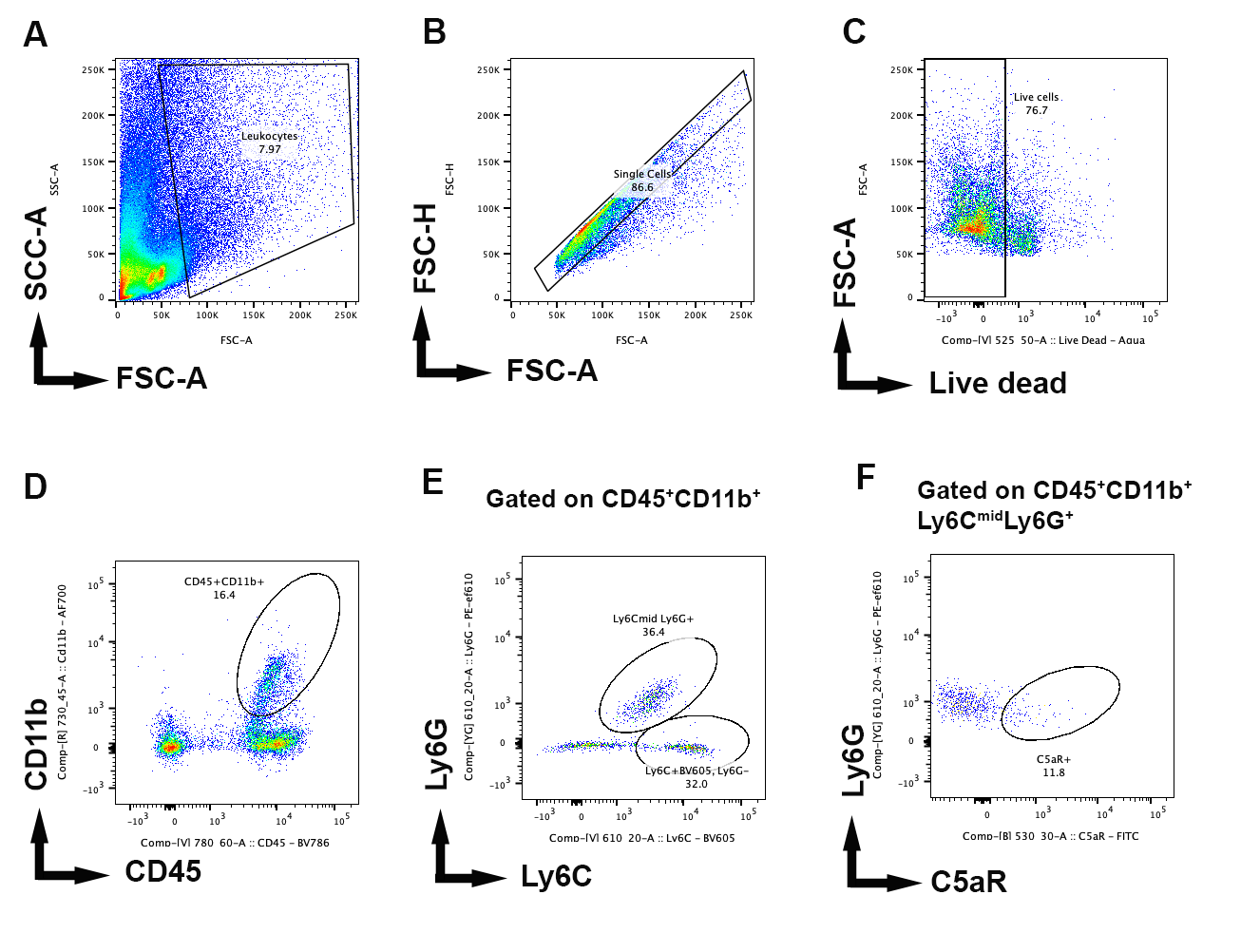

Supplement: Supplementary file 3 — Supplementary Figure 1: Gating Strategy for kidney flow cytometry. [file 41387_2024_305_MOESM3_ESM.tif]

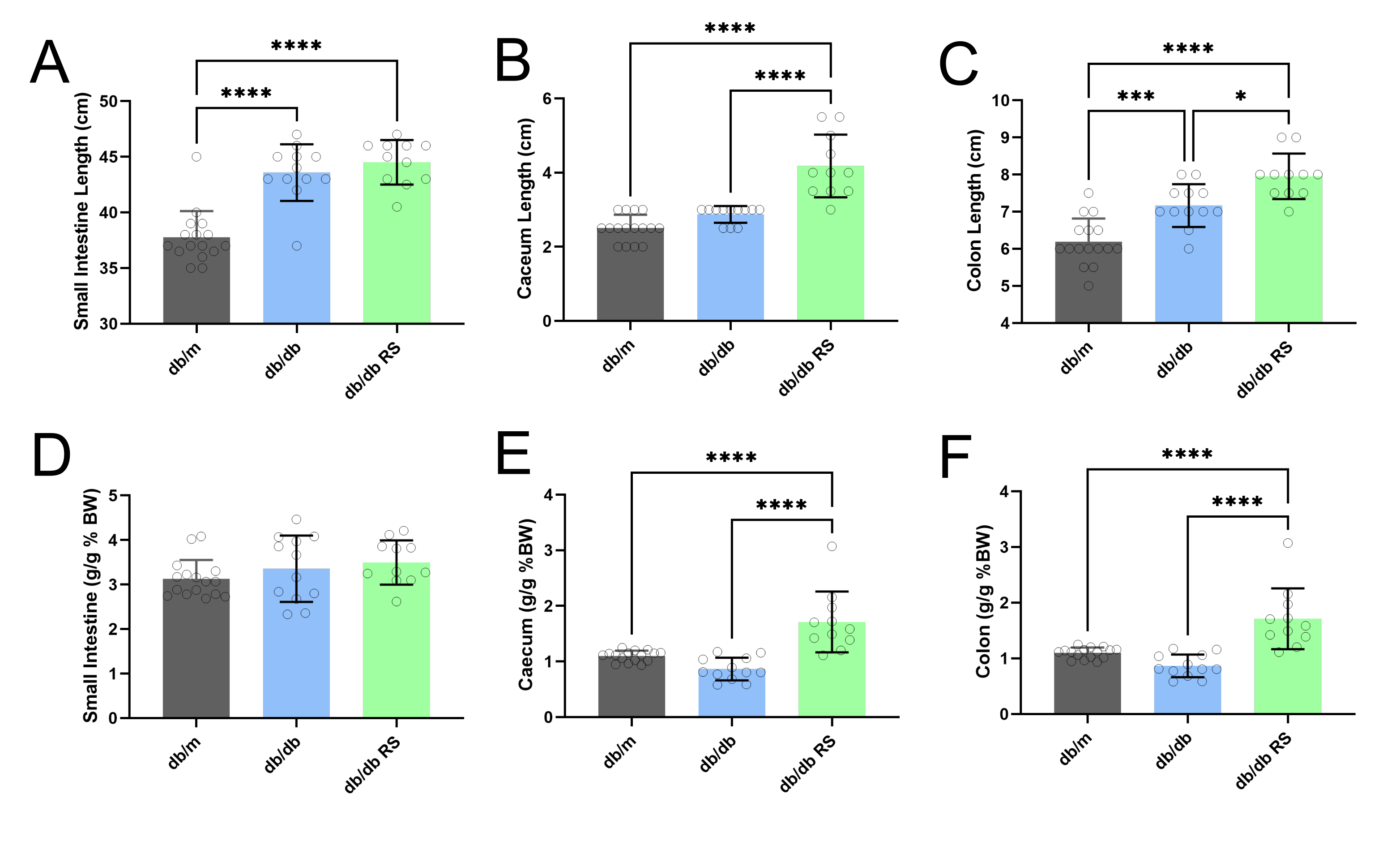

Supplement: Supplementary file 4 — Supplementary Figure 2: Resistant starch increases large intestinal, but not small intestinal, weight and length in diabetic mice. [file 41387_2024_305_MOESM4_ESM.png]
